# Sporulosol, a New Ketal from the Fungus *Paraconiothyrium sporulosum*

**DOI:** 10.3390/molecules23061263

**Published:** 2018-05-25

**Authors:** Chen Zhao, Peinan Fu, Yang Zhang, Xingzhong Liu, Fengxia Ren, Yongsheng Che

**Affiliations:** 1 State Key Laboratory of Toxicology & Medical Countermeasures, Beijing Institute of Pharmacology & Toxicology, Beijing 100850, China; lfyz34304@126.com (C.Z.); fpn1109@aliyun.com (P.F.); zhangyang@bmi.ac.cn (Y.Z.); 2 College of Pharmacy, Nanjing University of Chinese Medicine, Nanjing 210023, China; 3 State Key Laboratory of Mycology, Institute of Microbiology, Chinese Academy of Sciences, Beijing 100190, China; liuxz@im.ac.cn

**Keywords:** *Paraconiothyrium sporulosum*, ketal, racemate, cytotoxicity

## Abstract

Sporulosol (**1**), a new ketal, together with four known compounds, has been isolated from the liquid fermentation cultures of a wetland-soil-derived fungus, *Paraconiothyrium sporulosum*. Its structure was elucidated primarily by NMR experiments, and was further confirmed by X-ray crystallography. Sporulosol was obtained as a racemic mixture and the resolved two enantiomers racemized immediately after chiral separation. Sporulosol appears to be the first ketal derived from a 6*H*-benzo[*c*]chromen-6-one and a benzofuranone unit. The compound showed modest cytotoxicity toward the human tumor cell line T24, with an IC_50_ value of 18.2 µM.

## 1. Introduction

**Figure 1:**
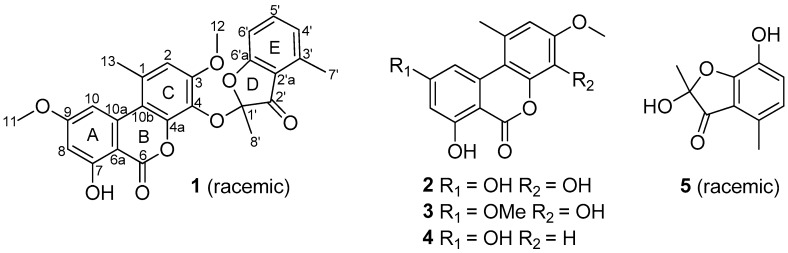
Structures of compounds **1**–**5**.

Fungi are one of the most important sources of new drugs or lead compounds; many important medicines have been discovered directly from fungi or developed based on their secondary metabolites [1], such as the antibiotic penicillin, anti-cholesteremic agent lovastatin, immuno-suppressant cyclosporin A, and the anti-multiple sclerosis fingolimod. Fungal species isolated from the wetland environments have attracted increasing attention due to their ability to produce a variety of bioactive secondary metabolites. Examples include stachybisbins A and B [2], flaviphenalenones A–C, aspulvinones P and Q, methybutyrolactone III [3,4], 5-hydroxymethylasterric acid, 3,5-dichlorosulochrin [4], and isocoumarin glycosides [5]. Based on this consideration, we initiated chemical studies of the wetland-soil-derived fungal species in the genus of *Paraconiothyrium*, which was reclassified as a separate genus as *Paraconiothyrium* by Verkley in 2004 [6]. Prior to our chemical studies, a variety of bioactive secondary metabolites were reported from this genus [7,8,9,10,11,12,13,14,15,16,17]. Our chemical investigations of *P*. *brasiliense*, and *P*. *hawaiiense* grown in solid-substrate fermentation cultures have also resulted in the isolation of structurally diverse and biologically active natural products including brasilamides A–N and hawaiinolides A–G [18,19,20,21,22]. During an ongoing search for new bioactive secondary metabolites from the species of this genus, a strain of *Paraconiothyrium sporulosum*, which was collected at Poyang Lake, Jiangxi Province, People’s Republic of China, was subjected to our chemical investigation. A new ketal, named sporulosol (**1**), was isolated from the liquid fermentation cultures of the fungus, together with four known compounds (**2**–**5**; Figure 1). Details of the isolation, structure elucidation, and cytotoxicity of these compounds are reported herein.

## 2. Results and Discussion

Sporulosol (**1**) was assigned a molecular formula of C_26_H_22_O_8_ (16 degrees of unsaturation) on the basis of high-resolution electrospray ionization mass spectrometry (HRESIMS). Analysis of its nuclear magnetic resonance (NMR) data (Table 1) revealed the presence of an exchangeable proton (*δ*_H_ 11.89), five methyl groups including two *O*-methyls, one ketal carbon (*δ*_C_ 105.4), 18 aromatic/olefinic carbons with six protonated, one carboxylic carbon (*δ*_C_ 164.4), and one α,β-unsaturated ketone carbon (*δ*_C_ 195.9). These data accounted for all the NMR resonances and suggested that **1** was a pentacyclic metabolite. The ^1^H and ^13^C NMR data of **1** (Table 1) revealed structural features that were closely related to the known compounds graphislactone A (**3**) [23] and 2-hydroxy-2,4-dimethyl-3(2*H*)-benzofuranone [24], but some chemical shifts for **1** were significantly different from those reported for the known compounds, warranting detailed 2D NMR analysis. Interpretation of the NMR data revealed a tetrasubstituted aryl ring (A), with a hydroxy and an *O*-methyl group attached to C-7 and C-9, respectively, which was confirmed by the HMBC correlations from H-8 to C-6a, C-7, and C-10a; H-10 to C-6a, C-9, and C-10a; OH-7 to C-7 and C-8; and from H_3_-11 to C-9. Correlations from H-2 to C-1, C-3, C-4, C-4a, and C-10b; H_3_-13 to C-1 and C-2; and from H_3_-12 to C-3 established the pentasubstituted aryl ring (C). In turn, the HMBC cross peaks from H-4′ to C-5′, C-6′, and C-7′; H-5′ to C-4′ and C-6′a; H-6′ to C-2′a and C-5′; and from H_3_-7′ to C-2′a, C-3′, and C-4′ enabled junction of the trisubstituted aryl ring (E) with a methyl group attached to C-3′. Additional correlations from H_3_-8′ to C-1′ and C-2′, plus the ^13^C NMR chemical shifts for C-2′a (*δ*_C_ 117.4) and C-6′a (*δ*_C_ 170.0), indicated that these three carbons form an α,β-unsaturated ketone with C-1′ connected to C-6′a via the C-1′/C-6′a ether linkage. Considering the ^1^H NMR chemical shift of OH-7 (*δ*_H_ 11.89) and the unsaturation requirement of **1**, the carboxylic carbon (*δ*_C_ 164.4) was connected to C-6, and acylated the C-4a oxygen to form ring B, while C-4 and C-1′ were attached to the only remaining oxygen atom to form an ester linkage. Based on these data, the planar structure of **1** was established.

**Figure 2:**
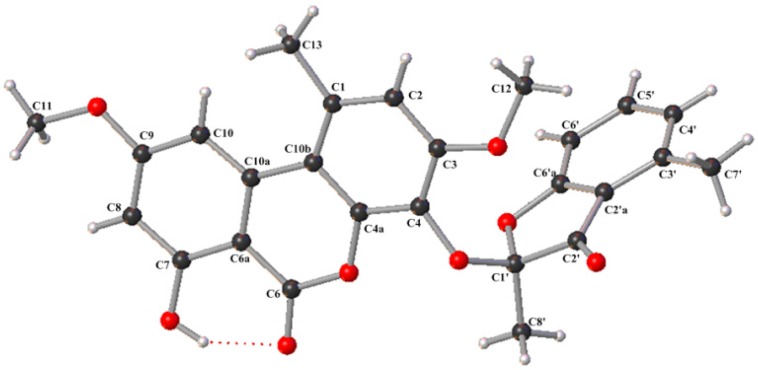
Thermal ellipsoid representation of **1.** (Note: A different numbering system is used for the structural data deposited with the CCDC.).

**Figure 3:**
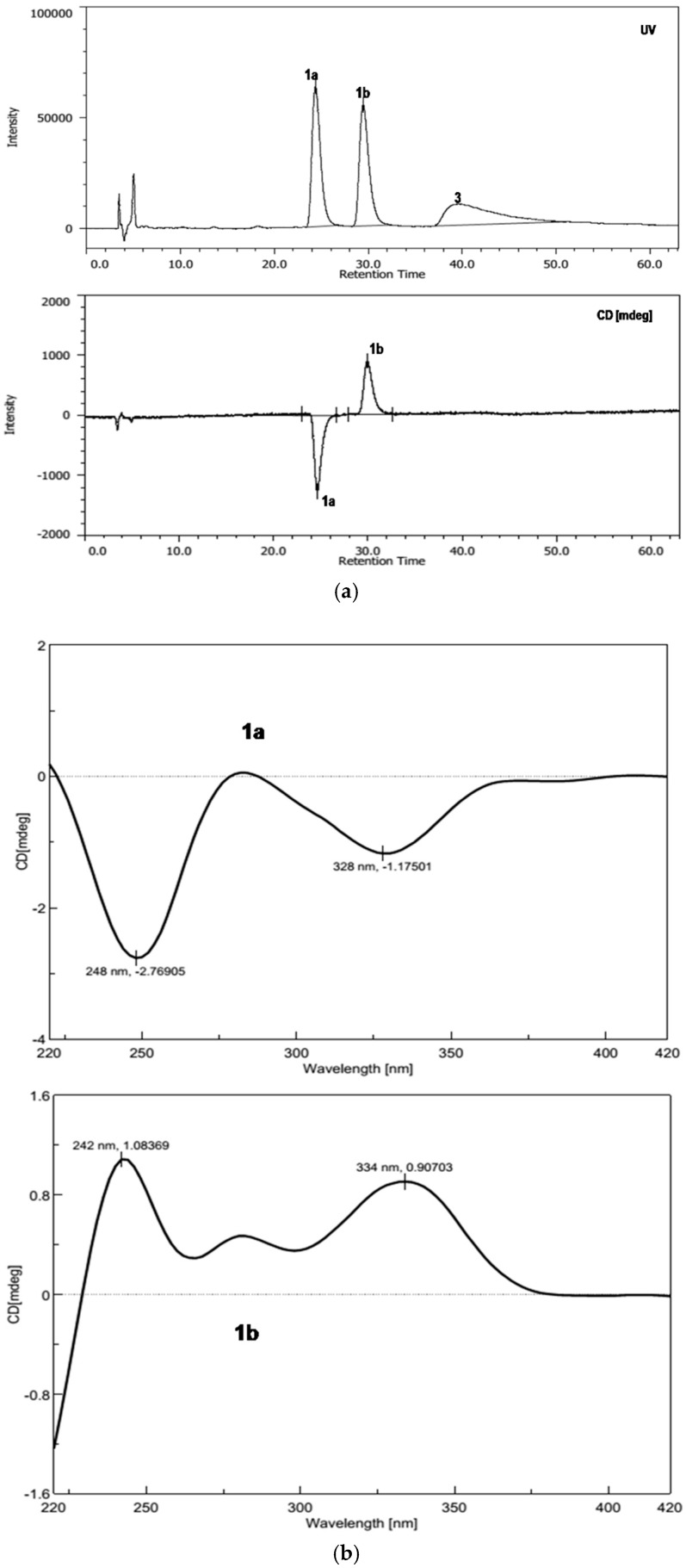
(**a**) HPLC-CD chromatogram of sporulosol (**1**) using a CHIRALPAK AD-H column (4.6 × 250 mm; 10% 2-Propanol in Hexane for 63 min; 1.0 mL/min); (**b**) HPLC-CD spectra of (1′*S*)-sporulosol (**1a**) and (1′*R*)-sporulosol (**1b**).

The proposed structure was confirmed by single-crystal X-ray diffraction analysis using Cu Kα radiation, and a perspective ORTEP plot is shown in Figure 2. Compound **1** was found to crystallize as a mixture of 1′*S* and 1′*R* enantiomers, which explained the low optical rotation value of +1.0 ([α]${}_{D}^{25}$; *c* 0.10, MeOH) measured for **1**. Subsequently, the racemic mixture was resolved into two enantiomers (**1a** and **1b**; 1:1) and decomposition product graphislactone A (**3**) using chiral stationary phase (4.6 × 250 mm; 4% 2-propanol in hexanes for 60 min; 0.8 mL/min) (Figure S7). However, the other decomposition product 2-hydroxy-2,4-dimethyl-3(2*H*)-benzofuranone was not detected during resolution. The absolute configuration of C-1′ was assigned by the application of Snatzke’s chirality rule for cyclopentenones [25], through which the negative or positive Cotton effect for *n* → *π* * transition in the 330–365 nm region of the CD spectrum was used to assign the 1′*S* (**1a**) and 1′*R* (**1b**) absolute configurations by HPLC-CD analysis (Figure 3). Analysis of each collected peak for 1′*S* (**1a**) and 1′*R* (**1b**) revealed the presence of both enantiomers, suggesting the occurrence of spontaneous equilibration. Similarly, the co-isolated known compound, enalin A (**5**) [26], was also separated into two enantiomers **5a** and **5b** in a ratio of 1:1, and the resolved enantiomers again racemized immediately after chiral separation (Figure S8). However, subsequent HPLC-CD analysis of **5** was unsuccessful, possibly due to the poor HPLC and CD behavior of the compound (Figure S9). To our knowledge, fungal natural products including isopestacin, pestacin, pestalachloride A, fimetarone A, and arugosins K−M, have been reported as racemic mixtures of the *S* and *R* enantiomers [27,28,29,30,31].

The molecular formula of **2** was determined to be C_15_H_12_O_6_ by HRESIMS. Analysis of the ^1^H and ^13^C NMR data of **2** (Table 1) revealed structural features similar to the known compound graphislactone A (**3**) [23], except that the 9-OMe signal disappeared, which was verified by interpretation of the 2D NMR data. Compound **2** was the first naturally occurring 4,7,9-trihydroxy-3-methoxy-1-methyl-6*H*-benzo[*c*]chromen-6-one [32], and its NMR data have yet to be reported. Compounds **3**–**5** were readily identified as graphislactone A (**3**) [23], 7,9-dihydroxy-3-methoxy-1-methyl-6*H*-benzo[*c*]chromen-6-one (**4**) [33], and enalin A (**5**) [26] by comparison of their NMR and MS data with those reported.

To verify that **1** was authentic natural product, a portion of the EtOAc extract freshly prepared from the freeze-dried fermented liquid substrate was subjected to HPLC-MS analysis using HPLC-grade H_2_O and CH_3_CN as solvents. Compound **1** was identified on the HPLC-MS chromatogram of the crude extract by comparison of its retention time and ESIMS data with an authentic sample (Figure S10), indicating that **1** is indeed a naturally occurring metabolite.

Compounds **1**–**4** were tested for cytotoxicity against a panel of five human tumor cell lines: HeLa, T24, A549, HCT116, and SH-SY5Y (Table 2). Compounds **1** and **4** showed modest cytotoxicity to T24 cells, with IC_50_ values of 18.2 and 4.5 µM, respectively (the positive control cisplatin showed an IC_50_ value of 10.9 µM). Compound **4** was also cytotoxic to Hela cells, showing an IC_50_ value of 5.4 µM (the positive control cisplatin showed an IC_50_ value of 8.7 µM).

**Scheme 1:**
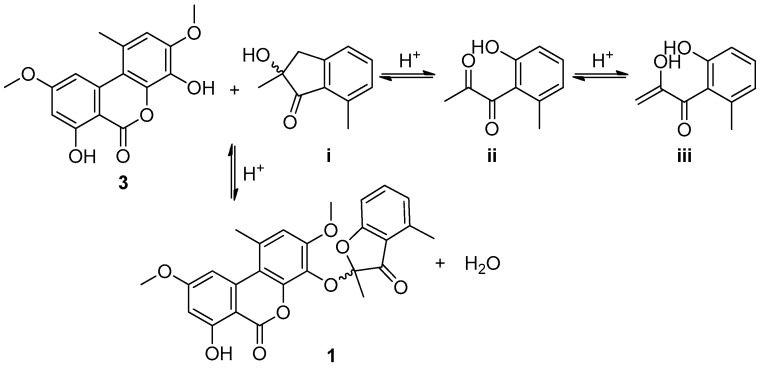
Plausible biosynthetic pathways for sporulosol (**1**).

Although sporulosol (**1**) is a ketal derived from two known metabolites, it is the first example of such a ketal originating from the 6*H*-benzo[*c*]chromen-6-one and benzofuranone moieties, representing a new type of chemical structure. The presence of such a ketal moiety generated two enantiomers racemized constantly even right after chiral resolution. In addition, the plausible biosynthetic precursor was identified as a racemic mixture, too. The proposed precursors and the reaction cascades leading to the generation of **1** are illustrated in Scheme 1.

## 3. Materials and Methods

### 3.1. General Experimental Procedures

An Analytical Automatic Polarimeter (Rodolph Research) was used to record optical rotations of the isolated compounds, and a Shimadzu Biospec-1601 spectrophotometer was used to measure the ultraviolet (UV) spectra. A Nicolet Magna-IR 750 spectrophotometer was used to record the infrared (IR) spectra. Nuclear magnetic resonance (NMR) spectra were recorded on Inova-400 and Inova-600 spectrometers using TMS as an internal standard. The HMBC and HMQC experiments were optimized for 8.0 and 145.0 Hz, respectively. Electrospray ionization mass spectrometry (ESIMS) and high-resolution mass spectrometry (HRMS) data were measured on an Agilent accurate mass quadrupole time-of-flight LC/MS G6550 instrument. High-performance liquid chromatography–circular dichroism (HPLC-CD) chromatograms were recorded on a JASCO LC 2000-CD 2095 instrument. All HPLC analysis and separation were performed using an Agilent 1260 instrument (Agilent, Santa Clara, CA, USA) equipped with a variable-wavelength UV detector.

### 3.2. Fungal Material

The strain *P. sporulosum* Verkley was isolated from the soil samples that were collected at Poyang Lake, Jiangxi Province, P. R. China, in December 2010. The fungus was identified by morphological observation and sequence (Genbank Accession No. JX077030) analyses of the ITS region of the rDNA. The identified *P. sporulosum* strain was cultured on Potato Dextrose Agar (PDA) at room temperature for 10 days, and the resulting agar plugs were cut into small pieces (0.5 × 0.5 × 0.5 cm^3^) under aseptic conditions. Fifteen pieces were inoculated into three 250 mL Erlenmeyer flasks, each containing 50 mL medium (0.4% glucose, 1% malt extract, and 0.4% yeast extract; pH 6.5), which were then incubated at room temperature on an orbital shaker at 170 rpm for 5 days to prepare the seed culture. The fermentation was carried out in 24 Fernbach flasks of 500 mL, each containing 5.0 mL seed culture and 200 mL synthetic dropout medium (2% malt extract, 6% dextrin, 0.7% peptone form fish, 0.7% cottonseed flour, 0.25% MgSO_4_·7H_2_O, 0.25% CaCO_3_, 0.1% FeSO_4_·7H_2_O, and 0.001% ZnSO_4_·7H_2_O), and incubated at 25 °C on a rotary shaker at 170 rpm for 30 days.

### 3.3. Extraction and Isolation

The fermented culture was extracted repeatedly with ethyl acetate (EtOAc; 4 × 4.8 L), yielding 5.0 g crude extract upon removal of the organic solvent under vacuum. Subsequently, the crude extract was fractionated by vacuum liquid chromatography on silica gel with gradient elution of petroleum ether (PE)–EtOAc. The fractions eluted with 88:12–82:18 PE–EtOAc were combined (338.4 mg) and separated by Sephadex LH-20 column chromatography (CC; 1:1 MeOH–CH_2_Cl_2_). The subfraction (119.5 mg) was purified by reversed-phase HPLC (Agilent Zorbax SB-C_18_ column; 5 μm; 9.4 × 250 mm; 45% MeOH in H_2_O for 38 min; 2 mL/min) to afford **5** (4.0 mg, *t*_R_ 21.0 min), and the resulting fraction eluted with 100% MeOH was further purified by RP HPLC (74% MeOH in H_2_O for 25 min; 2 mL/min) to afford **4** (1.0 mg, *t*_R_ 22.0 min). The fractions eluted with 80:20–78:22 PE–EtOAc were combined (445.0 mg) and separated by medium-pressure C18 RP silica gel CC using MeOH–H_2_O gradient elution (20–100%). Purification of the subfraction (68.9 mg) eluted with 80:20 MeOH–H_2_O by HPLC (70% MeOH in H_2_O for 15 min, followed by 82% MeOH in H_2_O for 30 min; 2 mL/min) yielded **3** (3.0 mg, *t*_R_ 16.5 min) and **1** (4.0 mg, *t*_R_ 41.0 min). The fractions eluted with 67:33–60:40 PE–EtOAc were combined (1.0 g) and further separated by C18 RP silica gel CC eluting with 20–100% MeOH–H_2_O. The subfractions eluted with 25:75–35:65 MeOH–H_2_O were combined (430.2 mg), separated by Sephadex LH-20 CC (1:1 MeOH–CH_2_Cl_2_), and purified by RP HPLC (55% MeOH in H_2_O for 40 min; 2 mL/min) to afford **2** (3.0 mg, *t_R_* 37.0 min).

### 3.4. Sporulosol *(**1**)*

Sporulosol (**1**), colorless needles; [α]${}_{D}^{25}$ +1.0 (*c* 0.10, MeOH); m.p. 130–132 °C; UV (MeOH) *λ*_max_ (log *ε*) 340 (3.57) nm; IR (neat) *ν*_max_ 3555, 2997, 2848, 1726, 1673, 1444, 1233, 1203, 1134 1078, 929, 795 cm^−1^; for ^1^H, ^13^C, and HMBC NMR data, see Table 1; HRESIMS *m*/*z* 463.1385 [M + H]^+^ (calcd. for C_26_H_22_O_8_, 463.1387).

X-ray Structure Analysis of **1** [34]. X-ray diffraction intensities were recorded with an Oxford Diffraction Gemini E diffractometer using Cu Kα radiation, *λ* = 1.5418, Ǻ at 99(6) K. All calculations were carried out using SHELXL-97 [35] and refined using full-matrix least-squares difference Fourier techniques. The Siemens Area Detector Absorption Program (SADABS) [36] was used to determine absorption corrections. The colorless crystal of **1** was obtained in acetone–H_2_O (30:1). Altogether, 4128 independent reflections were collected from the 10,139 measurements, yielding *R*_1_ = 0.0482 and *wR*_2_ = 0.1208 [*I* > 2*σ*(*I*)]. Crystal data: colorless crystal (0.20 × 0.18 × 0.04 mm); C_26_H_22_O_8_, *M* = 471.00, space group C2/c; monoclinic crystal; unit cell dimensions *a* = 32.3086(15) Ǻ, *b* = 8.9000(4) Ǻ, *c* = 15.4083(7) Ǻ, *V* = 4350.5(3) Ǻ^3^, *Z* = 8, *D*_calcd_ = 1.438 mg/mm^3^, µ = 0.906 mm^−1^, *F*(000) = 1974.

### 3.5. 4,7,9-Trihydroxy-3-methoxy-1-methyl-6H-benzo[c]chromen-6-one *(**2**)*

4,7,9-trihydroxy-3-methoxy-1-methyl-6*H*-benzo[*c*]chromen-6-one (**2**), white powder; UV (acetone) *λ*_max_ (log *ε*) 340 (2.20) nm; IR (neat) *ν*_max_ 3458, 2980, 2848, 2158, 1673, 1604, 1397, 1230, 1197, 1120, 798 cm^−1^; for ^1^H and ^13^C NMR data, see Table 1; HRESIMS *m*/*z* 289.0705 [M + H]^+^ (calcd. for C_15_H_12_O_6_, 289.0707).

### 3.6. MTT Assay

The cytotoxicity of compounds **1**–**4** was evaluated with the MTT assay [37]. The cell lines at a density of (2–5) × 10^3^ cells/well were seeded in 96-well plates and allowed to adhere for 24 h. Subsequently, compounds **1**–**4** and cisplatin were added at appropriate concentrations and incubated with cells at 37 °C for 48 h in a 5% CO_2_-containing incubator. Finally, 20 μL of MTS (Promega) was added to each well in the dark to assess the proliferation after 90 min incubation at 37 °C. The optical density was recorded on a microplate reader at 490 nm. All tests were run in triplicate.

## Figures and Tables

**Table 1:** NMR data for **1** and **2**.

Pos.	1			2		
	*δ*_C_*^a^*, Mult.	*δ*_H_*^b^* (*J* in Hz)	HMBC	*δ*_C_*^c^* Mult.	*δ*_H_*^d^* (*J* in Hz)	HMBC
1	134.0, qC			126.6, qC		
2	112.8, CH	6.90, s	1, 3, 4, 4a, 10b	112.8, CH	6.93, s	1, 3, 4, 10a, 10b, 12
3	153.8, qC			147.8, qC		
4	128.0, qC			140.6, qC		
4a	147.0, qC			138.8, qC		
6	164.4, qC			164.8, qC		
6a	98.9, qC			98.2, qC		
7	165.0, qC			165.0, qC		
8	99.5, CH	6.60, d (2.0)	7, 6a, 10a	101.3, CH	6.46, d (2.0)	6a, 9, 10
9	166.7, qC			165.3, qC		
10	104.2, CH	7.28, d (2.0)	6a, 9, 10a	104.9, CH	7.37, d (2.0)	6a, 8, 9, 10b
10a	137.9, qC			132.8, qC		
10b	111.0, qC			111.2, qC		
11	55.4, CH_3_	3.96, s	9	55.7, CH_3_	3.67, s	3
12	55.2, CH_3_	3.67, s	3	24.5, CH_3_	2.75, s	1, 2, 10, 10a
13	24.8, CH_3_	2.78, s	1, 2			
1′	105.4, qC					
2′	195.9, qC					
2′a	117.4, qC					
3′	139.8, qC					
4′	123.8, CH	6.88, d (7.8)	5′, 6′, 7′			
5′	137.7, CH	7.45, t (7.8)	4′, 6′a			
6′	109.8, CH	6.72, d (7.8)	2′a, 5′			
6′a	170.0, qC					
7′	16.7, CH_3_	2.53, s	2′a, 3′, 4′			
8′	19.4, CH_3_	1.80, s	1′, 2′			
OH-7		11.89, s	7, 8		11.95, s	6a, 7, 8

*^a^* Recorded at 100 MHz in acetone-*d*_6_; *^b^* Recorded at 400 MHz in acetone-*d*_6_; *^c^* Recorded at 150 MHz in acetone-*d*_6_; *^d^* Recorded at 600 MHz in acetone-*d*_6_.

**Table 2:** Cytotoxicity of compounds **1**–**4**.

Compound	IC_50_ (μM)				
	HeLa	T24	A549	HCT116	SH-SY5Y
**1**	31.8 ± 0.2	18.2 ±5.3	23.1 ± 2.9	50.1 ± 7.0	>50
**2**	11.7 ± 0.7	22.8 ± 1.7	25.1 ± 1.8	81.5 ± 3.4	41.5 ± 2.0
**3**	>50	77.8 ± 8.0	>50	>50	71.7 ± 3.2
**4**	5.4 ± 0.1	4.5 ± 0.4	93.7 ± 5.6	20.9 ± 2.6	38.8 ± 4.9
cisplatin	8.7 ± 0.3	10.9 ± 0.7	17.0 ± 0.5	19.9 ± 0.7	11.2 ± 2.0

## Supplementary Materials

The supplementary materials are available online. NMR spectra of Compounds **1** and **2** (Figures S1–S6), Figure S7: HPLC chromatogram of sporulosol (**1**) using a CHIRALPAK AD-H column; Figure S8: HPLC chromatogram of enalin A (**5**) using a CHIRALPAK AD-H column title; Figure S9: HPLC-CD chromatogram of enalin A (**5**) using a CHIRALPAK AD-H column title; Figure S10: HPLC-MS analysis of the crude extract.
