# Supplementary material for: Sporulosol, a New Ketal from the Fungus Paraconiothyrium sporulosum
Source: Molecules. 2018 May 25;23(6):1263. doi: 10.3390/molecules23061263 (PMC6100215; doi:10.3390/molecules23061263)

Sporulosol, a New Ketal from the Fungus *Paraconiothyrium sporulosum*

Chen Zhao [1], Peinan Fu [1,2], Yang Zhang [1], Xingzhong Liu [3], Fengxia Ren [1,*] and Yongsheng Che [1,*]

[1] State Key Laboratory of Toxicology & Medical Countermeasures, Beijing Institute of Pharmacology & Toxicology, Beijing 100850, China; lfyz34304@126.com (C.Z.); fpn1109@aliyun.com (P.F.); zhangyang@bmi.ac.cn (Y.Z.)
[2] College of Pharmacy, Nanjing University of Chinese Medicine, Nanjing 210023, China
[3] State Key Laboratory of Mycology, Institute of Microbiology, Chinese Academy of Sciences, Beijing 100190, China; liuxz@im.ac.cn
* Correspondence: renfx@bmi.ac.cn (F.R.); cheys@im.ac.cn (Y.C.); Tel.: +86-10-6693-2679 (Y.C.)

Contents **Page**

1) **Figure S1.** ^{1}H NMR spectrum of sporulosol (**1**; 400 MHz, acetone-d_6) 3

2) **Figure S2.** ^{13}C NMR spectrum of sporulosol (**1**; 100 MHz, acetone-d_6) 4

3) **Figure S3.** HSQC spectrum of sporulosol (**1**; 400 MHz, acetone-d_6) 5

4) **Figure S4.** HMBC spectrum of sporulosol (**1**; 400 MHz, acetone-d_6) 6

5) **Figure S5.** ^{1}H NMR spectrum of compound **2** (600 MHz, acetone-d_6) **7**

6) **Figure S6.** ^{13}C NMR spectrum of compound **2** (150 MHz, acetone-d_6) 8

7) **Figure S7.** HPLC chromatogram of sporulosol (**1**) using a CHIRALPAK AD-H column 9

8) **Figure S8.** HPLC chromatogram of enalin A (**5**) using a CHIRALPAK AD-H column 10

9) **Figure S9.** HPLC–CD chromatogram of enalin A (**5**) using a CHIRALPAK AD-H column 11

10) **Figure S10.** HPLC–MS analysis of the crude extract 12

Figure S1. ^{1}H NMR Spectrum of Sporulosol (**1**; 400 MHz, Acetone-d_6)

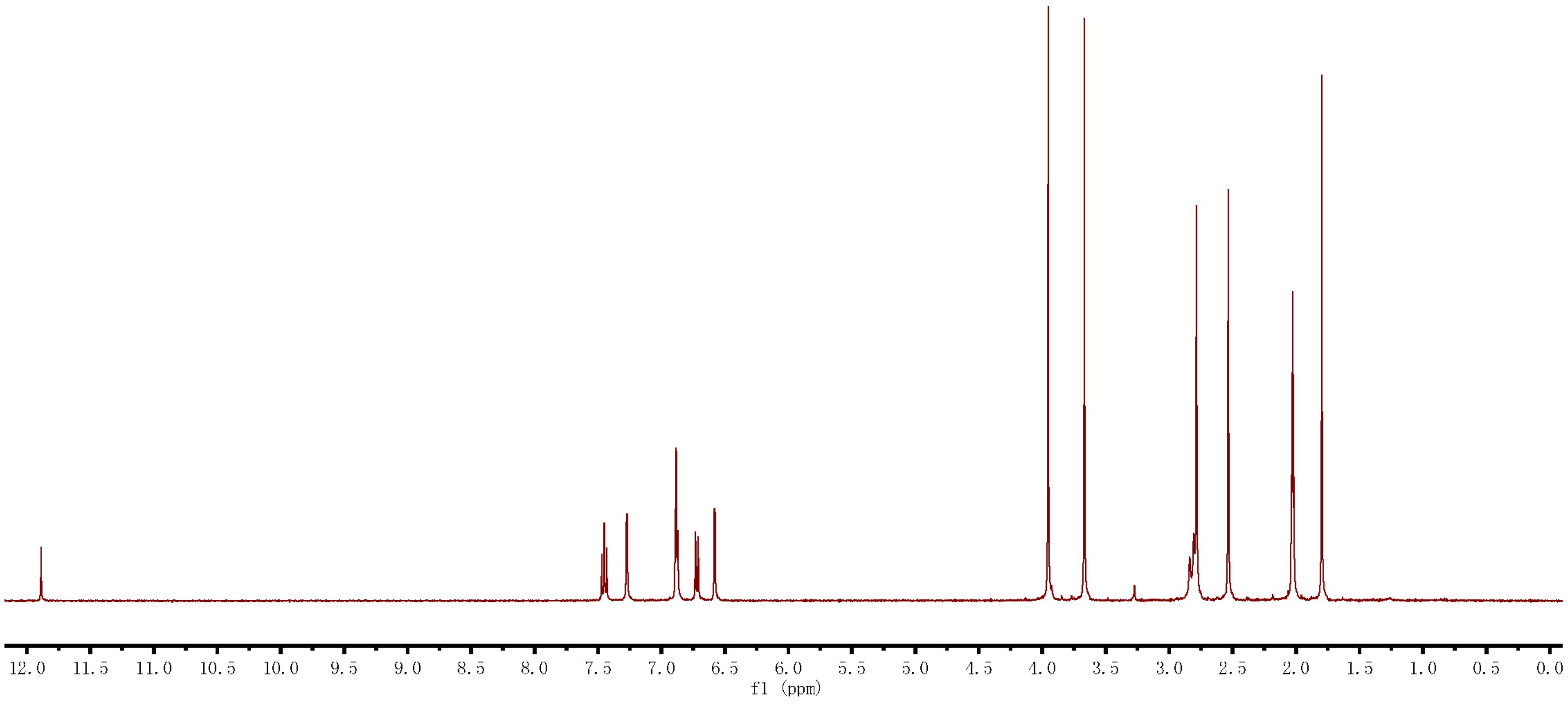

Figure S2. ^{13}C NMR Spectrum of Sporulosol (**1**; 100 MHz, Acetone-d_6)

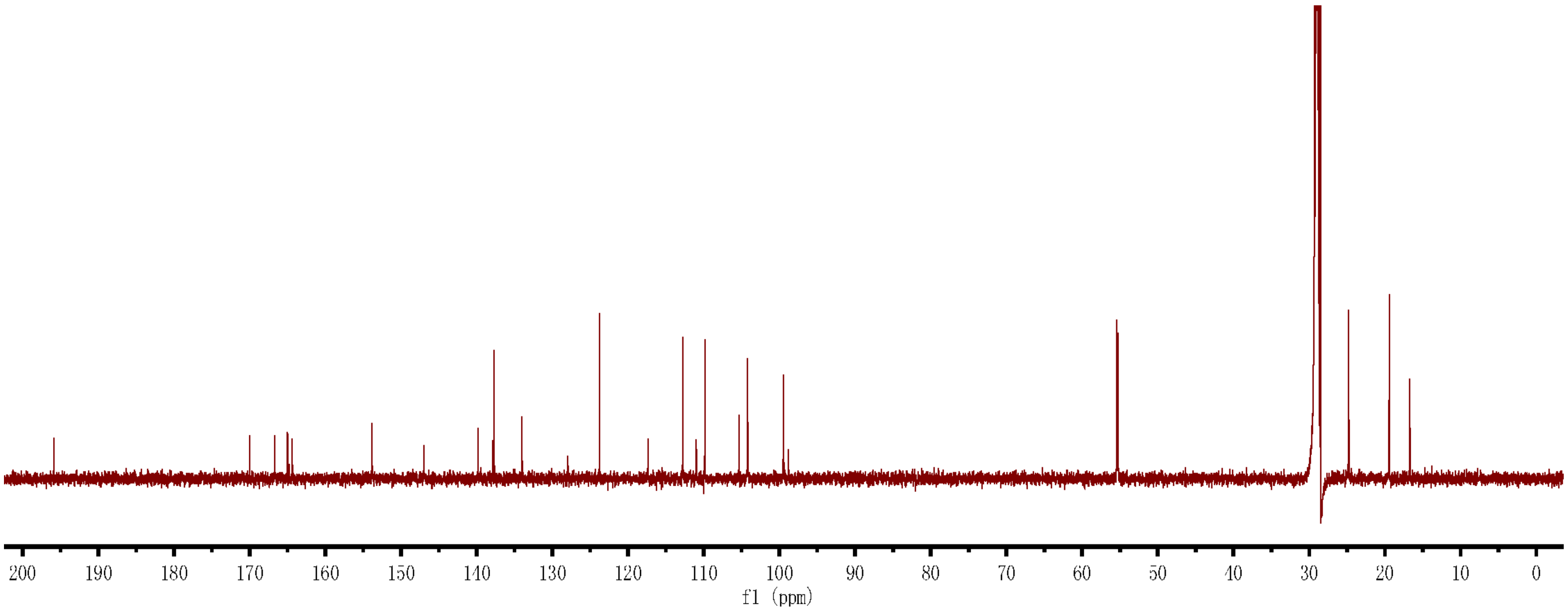

Figure S3. HSQC Spectrum of Sporulosol (**1**; 400 MHz, Acetone-d_6)

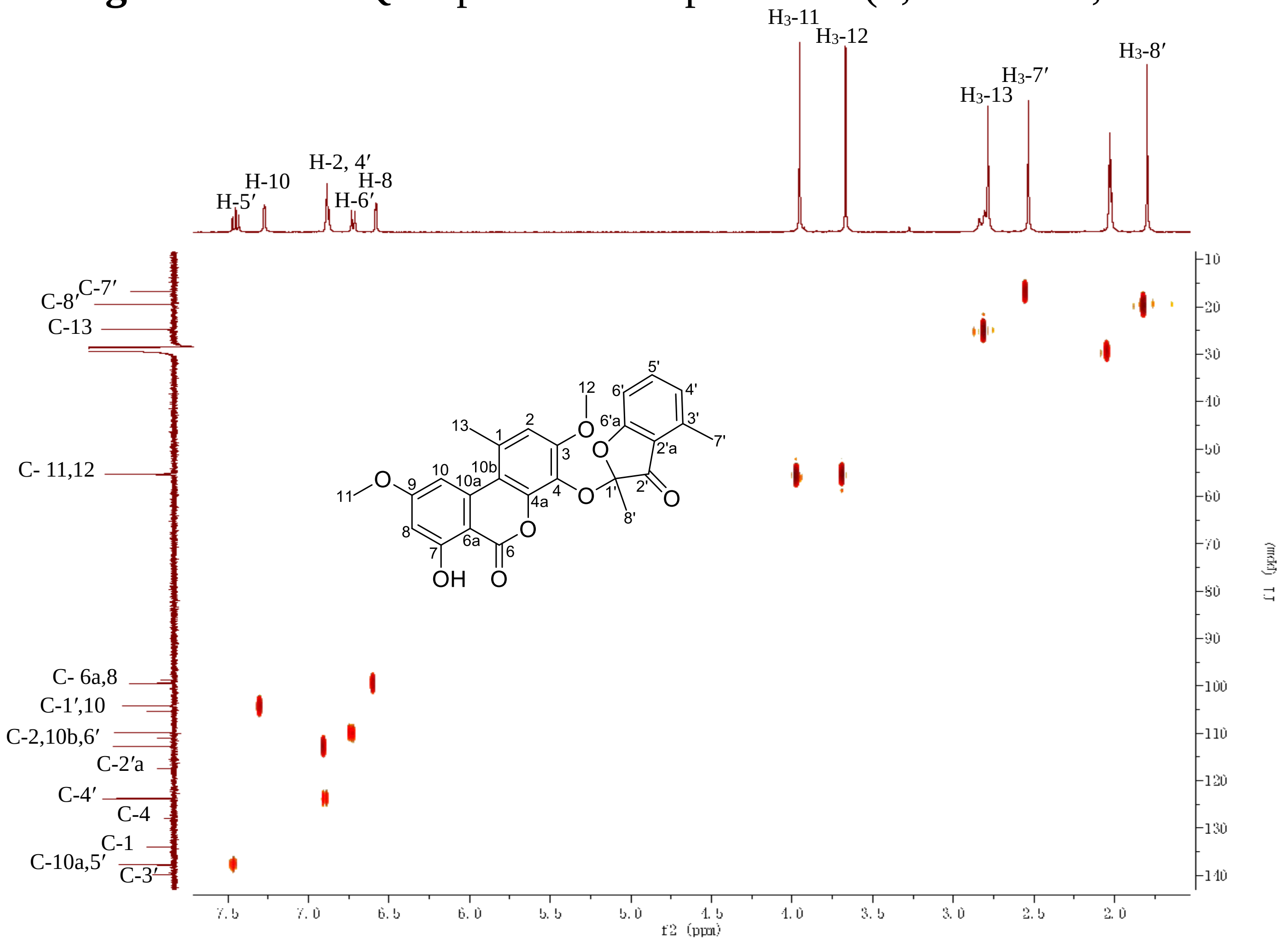

Figure S4. HMBC Spectrum of Sporulosol (**1**; 400 MHz, Acetone-d_6)

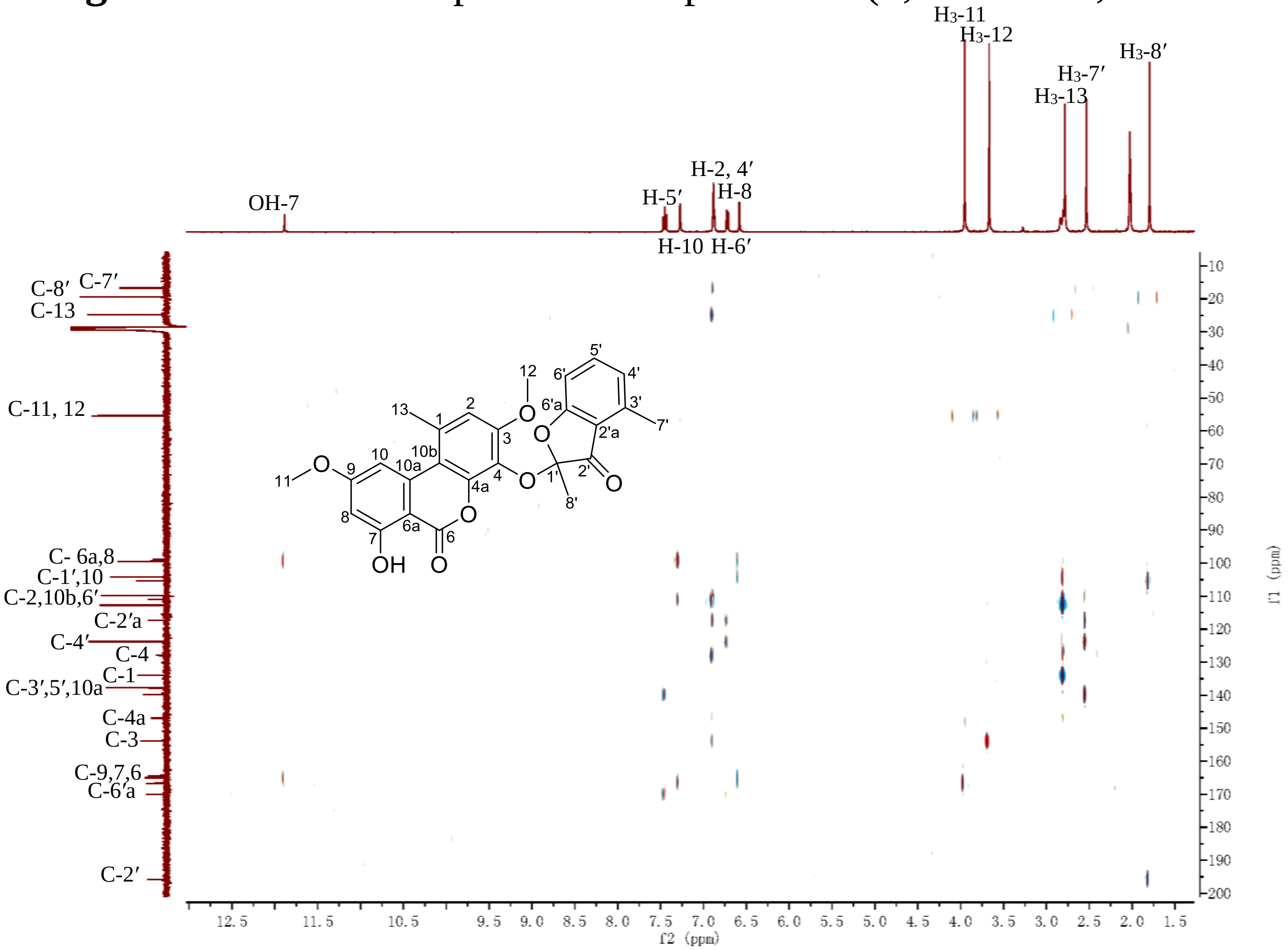

Figure S5. ^{1}H NMR Spectrum of Compound **2** (600 MHz, Acetone-d_6)

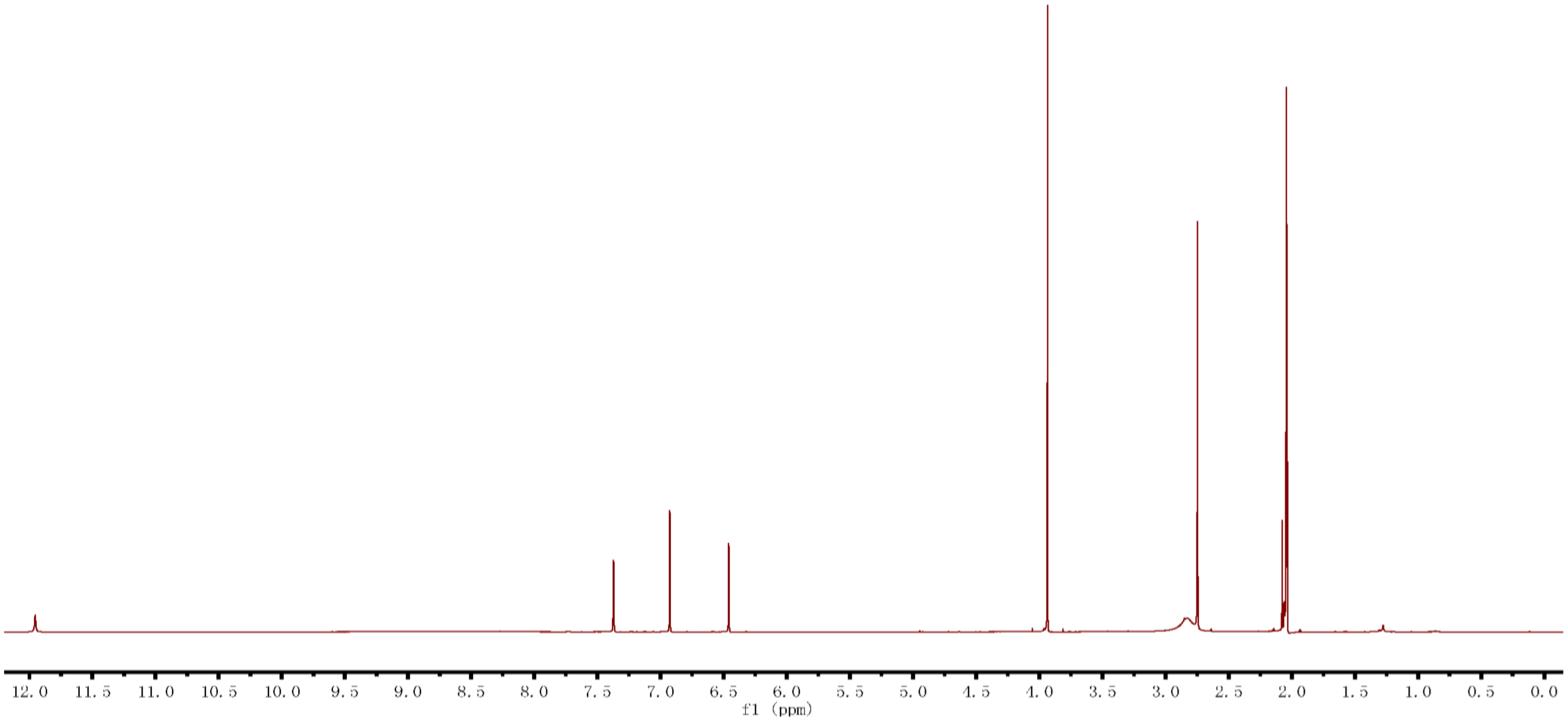

Figure S6. ^{13}C NMR Spectrum of Compound **2** (150 MHz, Acetone-d_6)

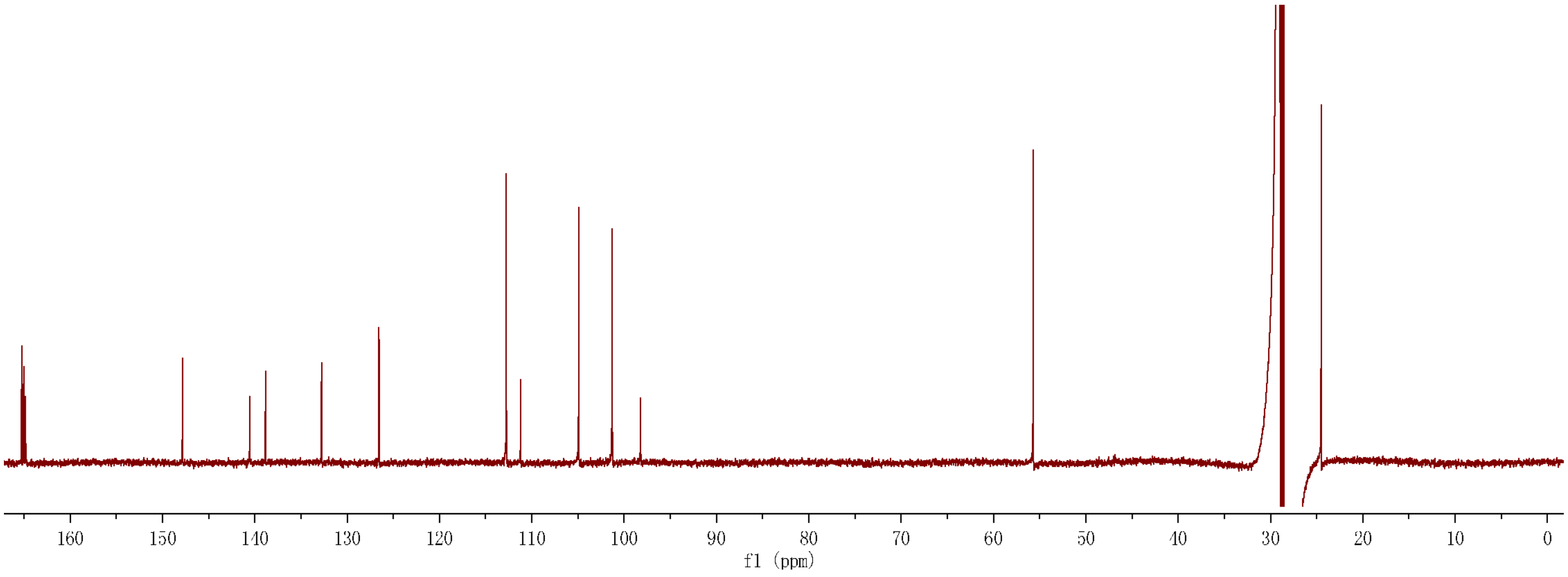

Figure S7. HPLC Chromatogram of Sporulosol (**1**) Using a CHIRALPAK AD-H Column

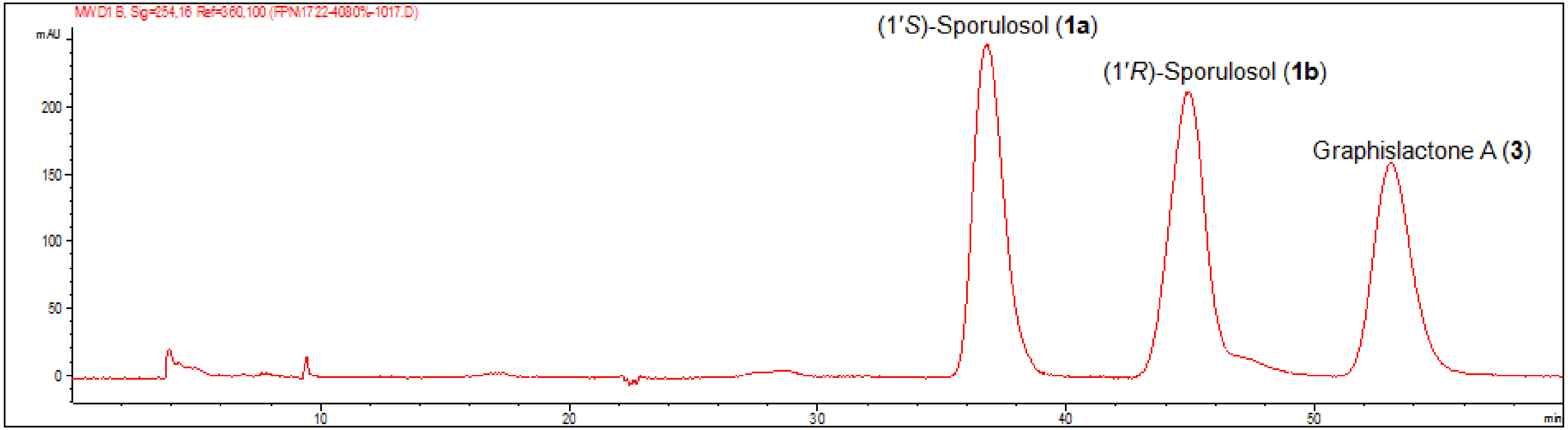

(4.6 × 250 mm; 4% 2-Propanol in Hexane for 60 min; 0.8 mL/min)

Figure S8. HPLC Chromatogram of Enalin A (**5**) Using a CHIRALPAK AD-H Column

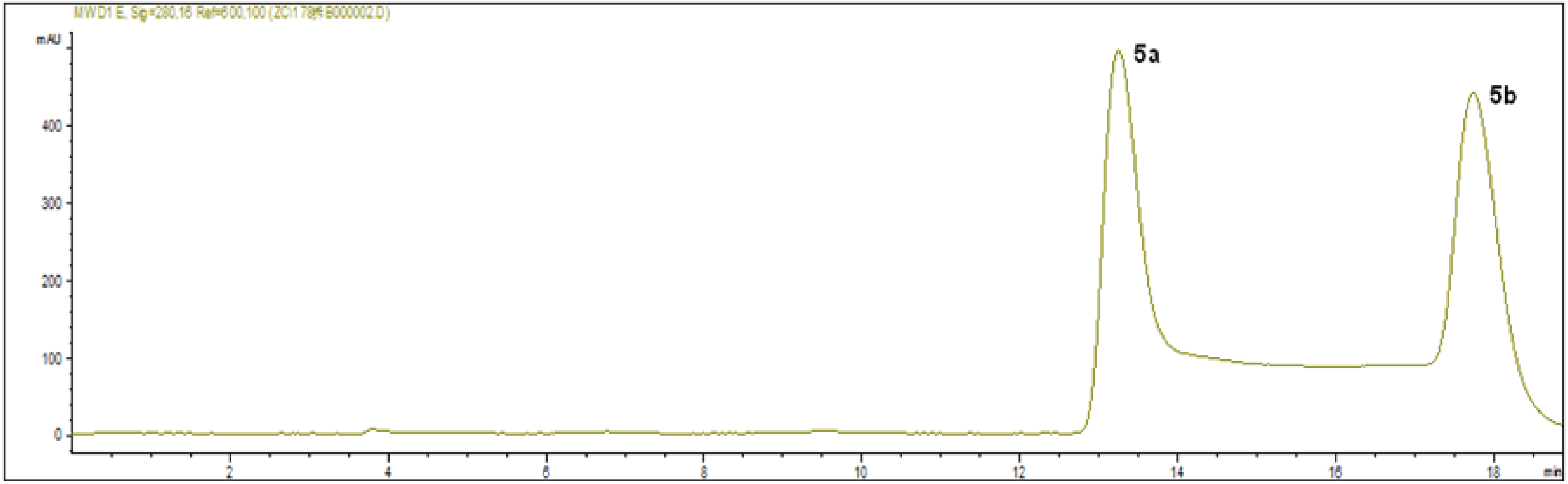

(4.6 × 250 mm; 6% 2-Propanol in Hexane for 20 min; 0.8 mL/min)

Figure S9. HPLC–CD Chromatogram of Enalin A (**5**) Using a CHIRALPAK AD-H Column

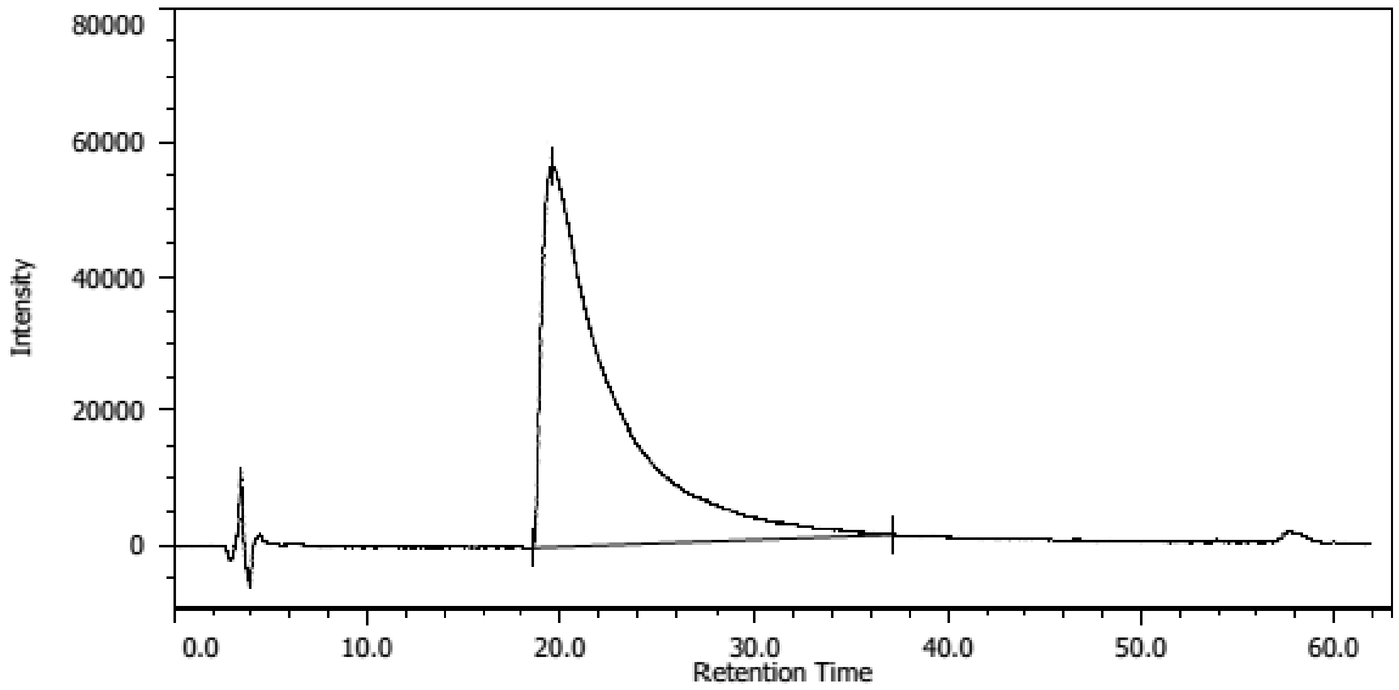

(4.6 × 250 mm; 8% 2-Propanol in Hexane for 63 min; 1.0 mL/min)

Figure S10. HPLC–MS Analysis of the Crude Extract

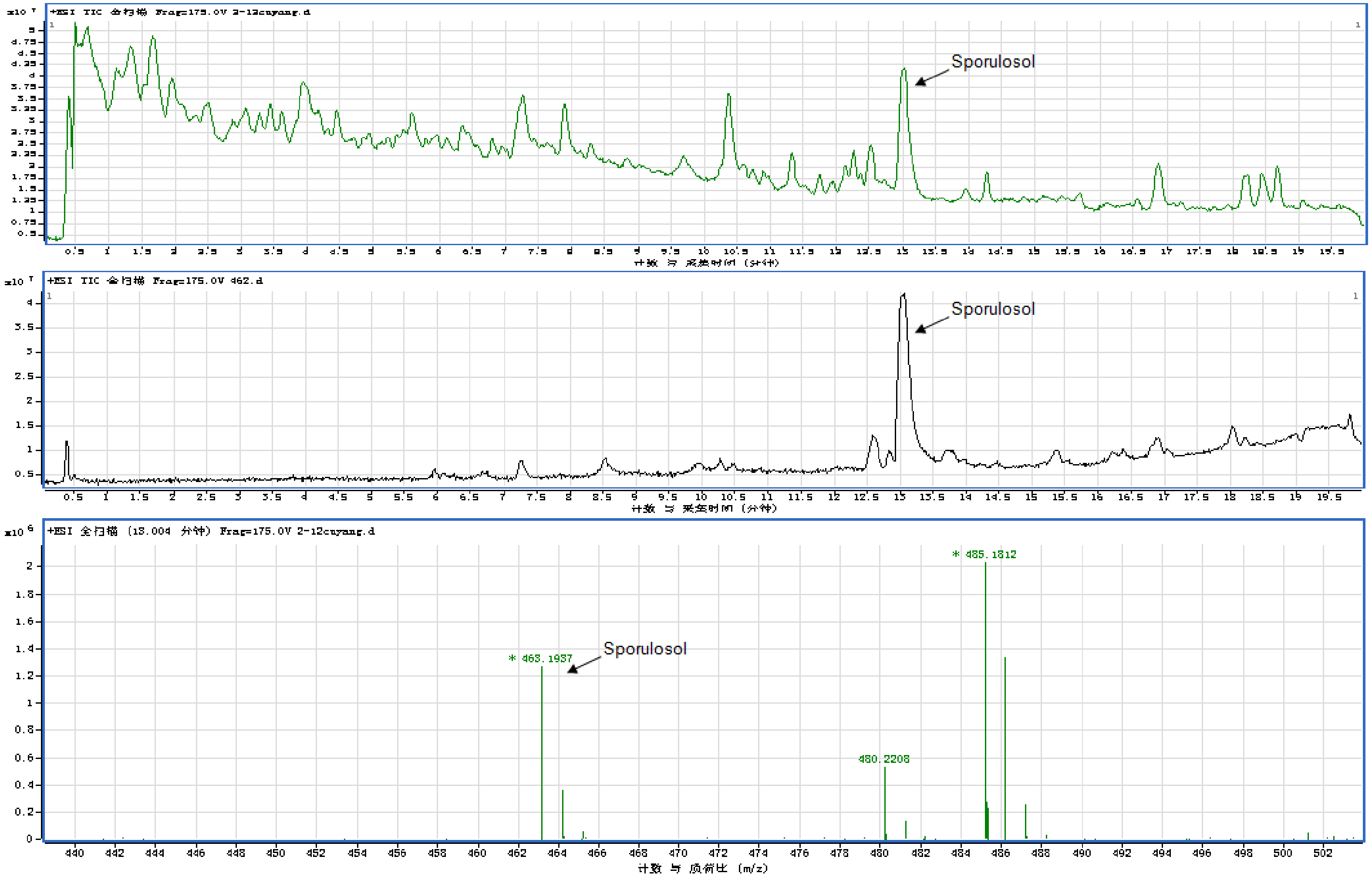

Supplement: Supplementary file 1 [file molecules-23-01263-s001.pdf]
